# Editorial: Cutting-Edge Nanotechnologies in Bacterial Infection Control

**DOI:** 10.3389/fchem.2022.942370

**Published:** 2022-06-08

**Authors:** Yong Liu, Fan Huang, Brandon W. Peterson, Qihui Zhou

**Affiliations:** ^1^ Wenzhou Institute, University of Chinese Academy of Sciences, Wenzhou, China; ^2^ China Academy of Chinese Medical Sciences, Beijing, China; ^3^ Department of Biomedical Engineering, University of Medical Center Groningen, Groningen, Netherlands; ^4^ Department of Stomatology, Institute for Translational Medicine, The Affiliated Hospital of Qingdao University, Qingdao University, Qingdao, China

**Keywords:** biofilm prevention, biofilm eradication, precise drug delivery, responsive nanocarriers, nanozymes

World Health Organization (WHO) has warned that multi-drug resistant bacteria will kill more people than cancer and be the primary killer by 2050 (World Health Organization, 2014), if we do not have effective solutions. Many factors contribute to the emergence of multi-drug resistant bacteria, such as the abuse/overuse of conventional antibiotics and bacteria living in biofilms or cells (Liu et al., 2021). Many nanoscale weapons have been established to combat multi-drug-resistant bacteria. Nanotechnology could facilitate the quantitative detection of bacterial infections, bacterial killing via a contact-kill manner, or drug delivery to overcome biological barriers.

In an original research article on this Topic, Lin et al. found that quercetin could be used as an efficient adjuvant to re-sensitize colistin against the colistin-resistant clinical isolates, such as *Escherichia coli* and *Klebsiella Pneumoniae.* Quercetin is efficient in downregulating the drug-resistance genes such as *mcr-1* and *mgrB* in those strains, suggesting that the combination of quercetin with colistin could provide an efficient alternative to the current clinical treatment for colistin-resistant pathogens.

In addition to adjuvants, modifying antibiotics via chemical methods is another effective way to strengthen the power of original antibiotics. Wu et al. summarized the recent methodologies applied to engineer polymyxin B for imaging and treatment purposes. Polymyxin B could be used as a building block to construct functional molecules or (nano)materials. Similar strategies can be easily expanded to other antibiotics, such as aminoglycosides and fluoroquinolones.

Qi et al. described the recent advances in nanomedicines to combat intracellular bacteria. Unlike their extracellular counterparts, intracellular pathogens are recalcitrant to antibiotic treatment and may induce chronic inflammation and autoimmune disorders, posing a severe threat to public health. Nanoscale weapons could assist in the targeting of infected cells and improve the bactericidal performance of conventional antimicrobials.

Besides bacterial infections, viruses and yeasts are also important microbes that cause diseases. To address those challenges, Pan et al. summarized that state-of-the-art research works had been applied to control human papillomavirus (HPV) infections. Nanoparticles could prevent the interactions between HPV and normal tissue cells and treat the virus-infected cells. Although the preclinical, experimental results were exciting, more samples and further study in the clinical translation are needed.

In treatments of *Candida albicans*-associated infections, Li et al. reviewed the recent nanocarriers such as inorganic nanoparticles, liposomes, polymeric nanoparticles, metal-organic frameworks, and covalent-organic frameworks that assist the penetration of antimicrobials in *Candida albicans* biofilms and their killing efficacy. Yet there is still a long way to go before these materials can be put into clinical use due to the low cost-effectiveness, potential cytotoxicity, etc.

Apart from the harmful pathogens to human health, pathogens that cause crop diseases are also covered in this topic. Gao et al. developed bismuth vanadate (BV) nanoparticles that can produce O_2_^−^ to eradicate common pathogens causing crop diseases such as *Thanatephorus cucumeris* and *Fusarium oxysporum*. BV is expected to be widely used in plant-related fields due to its excellent, low production cost, and antimicrobial properties.

Although various nanoscale weapons have been established for imaging bacterial infections, preventing bacterial adhesion and subsequent biofilm formation, and eradicating mature biofilms (Gupta et al., 2019; Liu et al., 2019), more efforts are stilled in dire need in the following aspects:

Firstly, a surface that prevents bacterial adhesion and subsequent biofilm formation usually does not allow the attachment and growth of mammalian cells as well. Therefore, technology that allows the tissue cells to win the “race to the surface” is highly desired.

Secondly, most of the current responsive nanosystems are responsive to only one stimulus. However, the progression of infections is closely associated with multi-physiological factors in the microenvironment, and designing multi-sensitive mechanisms would be helpful for fundamental studies and clinical applications.

Moreover, biodegradable nanosystems are needed to minimize the potential of cumulative cytotoxicity to the normal tissues. Also, more efforts to combine diagnosis and treatment of bacterial infections should be made.
